# Association of *TRPS1* gene with different EMT markers in ERα-positive and ERα-negative breast cancer

**DOI:** 10.1186/1746-1596-9-119

**Published:** 2014-06-16

**Authors:** Peng Su, Jing Hu, Hui Zhang, Ming Jia, Weiwei Li, Xuanxuan Jing, Gengyin Zhou

**Affiliations:** 1 Department of Pathology, Shandong University School of Medicine, 44#, Wenhua Xi Road, 250012 Jinan, Shandong, P.R. China; 2 Department of Pathology, Qilu Hospital of Shandong University, Jinan, PR China

**Keywords:** TRPS1, Epithelial to mesenchymal transition, Estrogen receptor, Breast cancer

## Abstract

**Background:**

Breast cancer is a heterogeneous disease consisting of different subtypes. Trichorhinophalangeal syndrome type 1 (*TRPS1*) gene, a GATA-type transcription factor, has been found to be highly expressed in breast cancer. Epithelial-to-mesenchymal transition (EMT) is known to play an important role in tumour invasion and metastasis. Our objective was to elucidate the different roles and clinical relevance of *TRPS1* in different estrogen receptor (ER) expression subtypes of breast cancer.

**Methods:**

An immunohistochemical study was performed. The correlation between clinicopathological features and other biomarker profiles were analysed statistically.

**Result:**

*TRPS1* expression was correlated with the patients’ age (P = 0.017). It was positively related with ERα (P < 0.001), progesterone receptor (PR) (P < 0.001) and ERβ (P = 0.001) status, but negatively associated with Ki67 (P = 0.002) and HER2 (P = 0.025) status. In ERα-positive breast cancer, *TRPS1* expression was positively associated with the expression of E-cadherin (P < 0.001), β-catenin(P = 0.001), ERβ (P = 0.03), and p53 (P = 0.002) status, while in ERα-negative breast cancer, *TRPS1* expression was correlated with slug (P = 0.004), vimentin (P = 0.003), smooth muscle actin (SMA) (P = 0.031), and IMP3 (P = 0.005) expression.

**Conclusions:**

Based on our findings, we conclude that *TRPS1* is positively associated with E-cadherin and β-catenin status in ERα-positive breast cancer cells, while it is also significantly associated with mesenchymal markers of EMT in ERα-negative breast cancer cells. *TRPS1* can be a prognostic marker depending on the type of breast cancer.

**Virtual Slides:**

The virtual slide(s) for this article can be found here: http://www.diagnosticpathology.diagnomx.eu/vs/8686515681264281

## Background

Breast cancer is one of the most common cancers in women, accounting for more than 1,300,000 cases and 450,000 deaths worldwide each year [1]. Breast cancer is a heterogeneous disease that comes in different morphological and immunohistochemical characteristics with corresponding clinical behaviour [2]. The oncogenesis and progression of breast cancer is a complex process involving a variety of transcription factors, activation of oncogenes, and inactivation of tumour suppressor genes [3,4].

Recently gene expression profiling and immunohistochemistry (IHC) studies have been identified Tricho-rhino-phalangeal syndrome-1 gene (*TRPS1*), a new GATA family member, to be highly prevalent gene in breast cancer [5,6]. TRPS is an autosomal dominant genetic disorder characterised by craniofacial and skeletal anomalies due to mutations or deletions of the *TRPS1* gene. It is composed of nine zinc finger motifs including a single GATA-type DNA-binding domain flanked by two potential nuclear localisation signals (NLS) and two C-terminal zinc fingers closely related to the domain found in the Ikaros family of lymphoid transcription factors [7]. It has been demonstrated that *TRPS1* is a transcriptional repressor and its activities are dependent on both the highly conserved GATA DNA-binding domain and the Ikaros-like zinc finger motifs [8]. For example, *TRPS1* can repress Stat3 to regulate proliferation and apoptosis of chondrocytes. *TRPS1* controls epithelial proliferation through repressing SOX9 in the developing vibrissa follicle in mice. It can also repress the expression of Runx2, a key regulator of osteoblastogenesis and chondrocyte maturation [9]. Moreover, *TRPS1* can suppress the osteocalcin expression through binding to its promoter [10].

As mentioned before, TRPS1 gene in human has been found to be overexpressed in breast cancer, expressed in more than 90% estrogen receptor α (ERα) positive and negative breast cancer subtype [6]. The gene is localised on human chromosome 8q23–24.1, a region highly amplified in several cancers, especially in prostate and breast cancer. It is important to note that *TRPS1* gene has been found to be highly expressed not only in the mammary glands but also in prostate, testis, ovaries, kidneys, and lungs [11]. Increasingly, there are more evidences to suggest the involvement of TRPS1 in a variety of functions in human cancers [12-15].

Recent studies have reported that *TRPS1* can regulate mesenchymal-to-epithelial transition (MET) during embryonic development in a number of tissues, including kidneys, cartilages, and bones [10,16,17]. Epithelial-to-mesenchymal transition (EMT) was first recognised as an important process during normal embryonic development [18]; however, carcinoma cells are also capable of reactivating EMT during tumour progression [19,20]. During this transition, tumour cells lose epithelial characteristics such as cell apical-basal polarity, membrane-associated adherents, and cell-to-cell adhesion protein E-cadherin. Concurrently, these tumour cells also undergo a dramatic remodelling of the cytoskeleton to facilitate cell mortality and invasion; the cells are also transformed to obtain a spindle-like phenotype. A key feature of EMT is a gene switch, resulting in downregulation of E-cadherin and upregulation of vimentin, smooth muscle actin. Transcriptional factors, such as snail, slug, and twist, which function by suppressing the expression of epithelial specific adhesion molecules, such as E-cadherin, were unveiled as key regulators inducing EMT in breast cancer and other cancers [21-24]. β-catenin was first identified as a protein that binds with E-cadherin to maintain cell-to-cell adhesion; however, it also functions as a transcription factor. Loss of membranous β-catenin expression and gain of cytoplasmic or nuclear β-catenin expression in neoplastic glands have been related to carcinogenesis and tumour progression in gastrointestinal cancers [25,26]. Thus, by detecting these EMT markers, one can roughly estimate the tumour cells undergoing EMT from non-EMT tumour cells.

In addition to the involvement of *TRPS1* in regulating MET, it has also been found to repress ZEB2, a key regulator of EMT that inhibits E-cadherin and other epithelial genes [12]. Realizing the potential of TRPS1 gene as the new EMT marker, we focused our work in elucidating different roles and clinical relevance of *TRPS1* in ERα-positive and ERα-negative breast cancer subtypes.

## Methods

### Patients and tissue samples

This study was conducted on 180 paraffin-embedded breast samples, which were histopathologically diagnosed invasive ductal carcinoma during 2007 to 2009 at the Department of Pathology of Qilu Hospital of Shandong University. For using these clinical materials for research purposes, prior patient consent and approvals from the Research Ethics Committee of Shandong Medical University were obtained. All the diagnoses were made following the Pathology and Genetics of Tumours of the Breast and Female Genital Organs of World Health Organisation Classification of Tumours.

### Tissue microarray

For each hematoxylin and eosin (H&E)-stained slide, two representative areas were selected and the corresponding spots were marked on the surface of the paraffin block. Using a tissue microarray (TMA) punching instrument, the selected areas were punched out and were placed into the recipient block side by side. Each tissue core was 2 mm in diameter and was assigned with a unique TMA location number that was linked to a database containing other clinicopathologic data [27].

### Immunohistochemistry

The immunohistochemical study was carried out to examine altered protein expression in 180 paraffin-embedded breast tissues as described in previous publications [28]. All the markers were incubated with the sections overnight at 4°C; the markers included *TRPS1* (sc-26974, diluted 1:200; Santa Cruz Biotechnology, CA, USA), P53 (Zhongshan Golden Bridge Biotechnology, ZSGB-Bio, Beijing, China), E-Cadherin (24E10, diluted 1:400, Cell Signalling Technology, USA), mouse monoclonal antibody β-catenin (E-5, diluted 1:500, Santa Cruz Biotechnology, CA, USA), Vimentin ( D21H3, diluted 1:100, Cell Signalling Technology, USA), slug (C19G7, diluted 1:50, Cell Signalling Technology, USA); the second antibody was from IHC reagent kit (Zhongshan Biotechnology Company, Beijing, China). After diaminobenzidine (DAB) staining, the sections were counterstained with hematoxylin. For negative controls, the antibodies were replaced with phosphate buffered saline (PBS).

### Evaluation of immunohistochemical staining

The stained slides were reviewed and evaluated independently by two observers blinded to patients’ information. A dual semi-quantitative scale, combining the staining intensity as well as percentage of positive cells, was used to evaluate the protein staining. In brief, staining of *TRPS1* was scored semi-quantitatively for intensity (0 = no expression, 1 = weak, 2 = moderate, and 3 = strong) and percentage of positive cells (0 = 0–10%, 1 = 10–30%, 2 = 30–50%; 3 = 50–80%, and 4 = 80–100%). The final score of TRPS1 was the staining score multiplied by the percentage of positive cells. The following cut-off levels were applied: 0 for negative and ≥ 1 for positive [15]. For β-catenin, membrane and cytoplasmic/nuclear expression were recorded separately as no staining, weak staining, or strong staining. Cases with more than 50% of nuclei stain were considered nuclear staining while cases with more than 50% of cytoplasm stain were considered cytoplasmic staining [29]. ER or PgR was positive if 1% of tumour cell nuclei were immuno reactive [30]. For other molecular markers, tumours were regarded as immune-positive if > 10% of tumour cells showed immunoreactivity. Cytoplasmic staining was considered positive for vimentin and SMA. Nuclear staining was considered positive for *TRPS1*, ERβ, slug and P53. Membranous staining was considered positive for E-cadherin.

### Statistical analysis

Statistical analyses were performed using the statistics software package SPSS 18.0 (SPSS, Chicago, IL). Chi-square test or Fisher’s exact test were performed to evaluate the correlation between *TRPS1* expression and clinicopathologic characteristics, if appropriate. Bivariate correlations between study variables were calculated by Spearman’s rank correlation coefficients. Differences were considered statistically significant for P values < 0.05.

## Results

### Relationship of *TRPS1* over-expression with the clinical features in breast cancer

In the analysis of a 180-member TMA, we found positive TRPS1 expression in 93 cases (51.7%), while 87 tumours (48.3%) were negative. Table 1 shows no significant correlation between the expression level of *TRPS1* and biological factors such as histology grade (P = 0.903), pathological stage (P = 0.646), tumour size (P = 0.343), lymph node metastasis (P = 0.443), P53 status (P = 0.113), and IMP3 status (P = 0.618). In contrast, we found that *TRPS1* expression was strongly correlated with the patients’ age (P = 0.017), Ki67 (P = 0.002), ERα (P < 0.001), progesterone receptor (PR) (P < 0.001), HER2 (P = 0.025), and ERβ status (P = 0.001). Spearman correlation analysis was preformed to confirm further the correlation between *TRPS1* expression and patients’ age (−0.179, P = 0.016); Ki67 (−0.233, P = 0.002), ERα (0.333, P < 0.001), PR (0.31, P < 0.001), HER2 (−0.166, P = 0.025), and ERβ status (0.242, P = 0.001). We divided the patients into two groups as ERα-positive and ERα-negative. *TRPS1* expression was observed in 69 (65.7%) of 105 ERα-positive patients, while in 24 (32%) of 75 ERα-negative patients.

**Table 1:** Correlation between TRPS1 expression and the clinicopathologic characteristics of breast cancer patients

**Characteristics**	**n**	**TRPS1 expression**		***P *** **value**	**Spearman**	**Value (r)**	***P *** **value**
		**Negative**	**Positive**		**Correlation**		
Age(y)							
≤50	70	26	44	0.017		−0.179	0.016
>50	110	61	49				
Grade							
I	11	5	6	0.903			
II	124	59	65				
III	45	23	22				
Pathological stage							
I	49	23	26	0.646			
II	85	44	41				
III	46	20	26				
Tumor size							
<2	60	26	34	0.343			
≥2	120	61	59				
Lymph node metastasis							
Negative	94	48	46	0.443			
Positive	86	39	47				
Expression of Ki67							
≤25%	62	20	42	0.002		−0.233	0.002
>25%	118	67	51				
Expression of P53							
Negative	72	40	32	0.113			
Positive	108	47	61				
ERα							
Negative	75	51	24	<0.001		0.333	<0.001
Positive	105	36	69				
PR							
Negative	75	50	25	<0.001		0.31	<0.001
Positive	105	37	68				
HER-2							
Negative	124	53	71	0.025		−0.166	0.025
Positive	56	34	22				
E-cadherin							
Negative	33	24	9	0.002		0.231	0.002
Positive	147	63	84				
Slug							
Negative	170	86	84	0.019		0.186	0.012
Positive	10	1	9				
Vimentin							
Negative	165	84	81	0.022		0.171	0.022
Positive	15	3	12				
SMA							
Negative	169	85	84	0.039		0.154	0.039
Positive	11	2	9				
β-catenin							
Negative	67	42	25	0.003		0.221	0.003
Positive	113	45	68				
ERβ							
≤2	92	55	37	0.001		0.242	0.001
>2	87	31	56				
IMP3							
Negative	157	77	80	0.618			
Positive	23	10	13				

### Relationship of *TRPS1* expression with EMT markers in breast cancer

As *TRPS1* may be a critical regulator of EMT during breast cancer initiation and progression, the expression of EMT markers, including E-cadherin, β-catenin, vimentin, SMA and slug, were stained in human breast cancer TMA. Statistical analysis indicated that the immunohistochemical expression of *TRPS1* is directly correlated with E-cadherin (P = 0.002) and β-catenin (P = 0.003), which was further confirmed by Spearman correlation analysis (E-cadherin: r = 0.231, P = 0.002; β-catenin: r = 0.221, P = 0.003) (Table 1). Moreover, we also found that *TRPS1* expression was significantly correlated with slug (P = 0.019), vimentin (P = 0.022) and SMA (P = 0.039); the corresponding Spearman correlation values are 0.186 (P = 0.012), 0.171 (P = 0.022), and 0.154 (P = 0.039).

### Correlation between *TRPS1* expression and molecular markers in ERα-positive breast cancer

**Figure 1:**
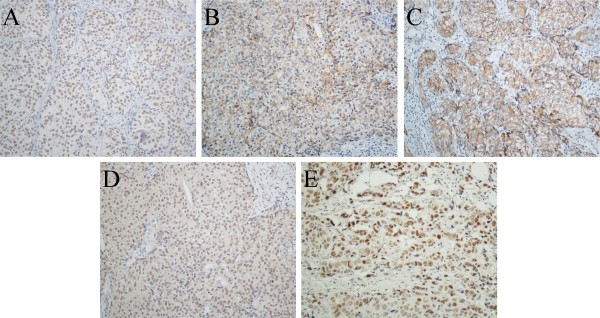
**Expression of markers in ERα-positive breast cancer (Magnification 200X). A**. Positive nuclear staining for *TRPS1*, **B**. Positive membranous staining for E-cadherin, **C**. Positive membranous staining for β-catenin, **D**. Positive nuclear staining for ERβ, and **E**. Positive nuclear staining for P53.

We analysed the molecular markers in ERα-positive breast cancer. Table 2 shows the expression of *TRPS1* is positively associated with E-cadherin (P < 0.001), β-catenin (P = 0.001), ERβ (P = 0.03), and p53 (P = 0.002) status (Figure 1). However, the *TRPS1* expression is not associated with slug (P = 0.549), vimentin (P = 0.296), SMA (P = 0.296), and IMP3 (P = 0.605).

**Table 2:** **Correlation between TRPS1 expression and molecular markers in ER**α **positive breast cancer patients**

**Characteristics**	**n**	**TRPS1 expression**		***P *** **value**	**Spearman**	**Value (r)**	***P *** **value**
		**Negative**	**Positive**		**Correlation**		
Age(y)							
≤50	42	9	33	0.023		−0.221	0.023
>50	63	27	36				
	Expression of P53						
Negative	45	23	22	0.002		0.307	0.001
Positive	60	13	47				
	E-cadherin						
Negative	16	13	3	<0.001		0.42	<0.001
Positive	89	23	66				
Slug							
Negative	102	36	66	0.549			
Positive	3	0	3				
Vimentin							
Negative	101	36	65	0.296			
Positive	4	0	4				
SMA							
Negative	101	36	65	0.296			
Positive	4	0	4				
β-catenin							
Negative	34	19	15	0.001		0.315	0.001
Positive	71	17	54				
ERβ							
≤2	46	21	25	0.03		0.211	0.03
>2	59	15	44				
IMP3							
Negative	101	34	67	0.605			
Positive	4	2	2				

### Correlation between *TRPS1* expression and molecular markers in ERα-negative breast cancer

**Figure 2:**
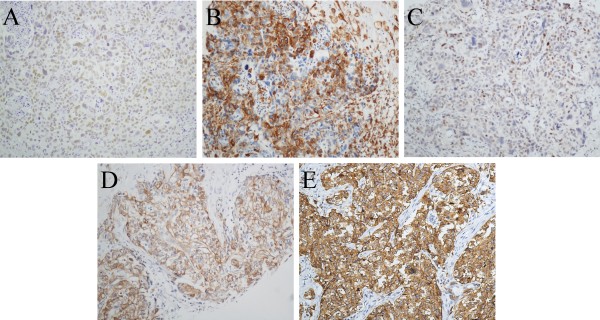
**Expression of markers in ERα-negative breast cancer (Magnification 200X). A**. Positive nuclear staining for *TRPS1*, **B**. Positive cytoplasmic staining for vimentin, **C**. Positive nuclear staining for slug. **D**. Positive cytoplasmic staining for SMA, and **E**. Positive cytoplasmic staining for IMP3.

For ERα negative breast cancer cases, Table 3 shows that the immunohistochemical expression of *TRPS1* has correlation with slug (P = 0.004), vimentin (P = 0.003), SMA (P = 0.031), and IMP3 (P = 0.005), which was further confirmed by Spearman correlation analysis (Figure 2).

**Table 3:** **Correlation between TRPS1 expression and molecular markers in ER**α **negative breast cancer patients**

**Characteristics**	**n**	**TRPS1 expression**		***P *** **value**	**Spearman**	**Value (r)**	***P *** **value**
		**Negative**	**Positive**		**Correlation**		
Age(y)							
≤50	28	17	11	0.296			
>50	47	34	13				
	Expression of P53						
Negative	27	17	10	0.483			
Positive	48	34	14				
	E-cadherin						
Negative	17	11	6	0.741			
Positive	58	40	18				
Slug							
Negative	68	50	18	0.004		0.369	0.001
Positive	7	1	6				
Vimentin							
Negative	64	48	16	0.003		0.362	0.001
Positive	11	3	8				
SMA							
Negative	68	49	19	0.031		0.271	0.019
Positive	7	2	5				
β-catenin							
Negative	33	23	10	0.78			
Positive	42	28	14				
ERβ							
≤2	46	34	12	0.167			
>2	29	17	12				
IMP3							
Negative	56	43	13	0.005		0.323	0.005
Positive	19	8	11				

## Discussion

Breast cancer is a heterogeneous disease consisting of multiple molecular subtypes. The presence of hormone receptors ER, PR, and human EGFR-2 (HER-2) are significantly meaningful in therapeutic decision-making for patients with breast cancer. In addition, these factors may also predict the probability of disease relapse. Hormone receptor-positive tumours have favourable outcomes because of their response to endocrine manipulations such as tamoxifen, aromatase inhibitors, or ovarian ablation [31]. Tumours with ERα-negative phenotype tend to have poor prognosis, unlike their hormone receptor or HER-2 positive counterparts; hence, such ERα-negative tumours lack targeted therapeutics.

Next, we investigated the expression of *TRPS1* in primary human breast cancer samples and explored its association with major breast tumour histological specialties and patients’ clinical characteristics. *TRPS1* was positively associated with ER and PR expressions and negatively associated with HER2 status, which is consistent with previous study [6].

When the cut-off level score for TRPS1 to be positive is 1, the TRPS1 was not remarkably associated with the tumour grade, pathological stage, tumour size and lymph node metastasis. However, upon changing the cut-off level score of *TRPS1* to be positive as ≥ 2, *TRPS1* was found to be positively associated with lymph node metastasis and P53 status (data not shown).

However, previous studies showed that higher *TRPS1* expression, when analysed using univariate and multivariate models, predicted better overall survival (OS) and disease-free survival (DFS) in a subgroup of ERα+, stage I/II breast cancer patients who received endocrine therapy only [32]. Recent studies have also confirmed that high *TRPS1* expression was significantly associated with lymph node metastasis and higher pathological stage of patients with colon cancer [14]. All these findings support our hypothesis that *TRPS1* may not merely be an indicator of better prognosis as shown in other studies. However, further research using larger patient cohort and more breast cancer cell lines is required to elucidate this contradictory result.

Epithelial cancer cells attain mesenchymal features that make them easier to invade the surrounding tissues and metastasise during EMT process. We used IHC to analyse these markers in 180 patients. Although *TRPS1* was significantly associated with E-cadherin and β-catenin, it was also positively associated with mesenchymal markers such as vimentin, SMA, and slug. The above data did not comply with previous results that *TRPS1* inhibits EMT process in breast cancer progression [12]. Hence, we divided the patients into two groups as ERα-positive and ERα-negative and reanalysed these EMT markers. Surprisingly, in ERα-positive breast cancer, we found *TRPS1* to be positively associated with E-cadherin and β-catenin status with no significant correlation with any of the mesenchymal markers. Consistent with our hypothesis, significant association was also found between *TRPS1* expression and E-cadherin expression in ERα + breast cancer cases [32]. In ERα-negative breast cancer, we found *TRPS1* to be positively associated with vimentin, SMA and slug. *TRPS1* was also found to be positively related with IMP3, which is expressed preferentially in triple negative breast cancers (TNBC). IMP3 is a member of insulin-like growth factor II (IGF-II) mRNA-binding proteins family. It contributes to the migration and invasion of TNBC cells. Thus, *TRPS1* may be associated with the migration, invasion, and EMT in ERα-negative breast cancer cells. Ligand-activated ERα could suppress slug transcription through direct association with the slug promoter. Human breast cancers, which lack ligand-activated ERα, may then over-express slug that may downregulate E-cadherin and lead to EMT [33]. We found *TRPS1* to be associated with different EMT markers by different ERα status, therefore, ERα might play an important role in affecting the relationship between *TRPS1* and EMT markers.

Estrogen receptors include estrogen receptor α (ERα) and estrogen receptor β (ERβ). Most studies have provided evidence that ERβ acts as a negative modulator of ERα and indicates a good prognosis with prolonged DFS [34,35]. Several investigators have found ERβ expression to be positively correlated with poor prognostic phenotypes such as accelerated proliferation and basal phenotype in ERα-negative breast cancer [36,37]. We also stained ERβ and found its expression to be correlated with *TRPS1* expression. *TRPS1* expression was not regulated by ER signalling since estrogen withdrawal using charcoal-stripped serum did not affect *TRPS1* gene or protein expression in ERα-positive breast cancer cell lines [32]. Thus, we have assumed that *TRPS1* might transcriptionally both regulate genes and affect tumour growth and development to varying degrees.

## Conclusions

There are many possible explanations for the different association between the molecular markers with *TRPS1* in ERα-positive and ERα-negative breast cancer. First, *TRPS1* is capable of binding to numerous cofactors of various functions. Second, it may either promote or inhibit carcinoma processing depending on the context and amount of protein present [38,39]. Based on the above results, we conclude that *TRPS1* is positively associated with E-cadherin and β-catenin status in ERα-positive breast cancer, while it also has a significant association with mesenchymal markers of EMT in ERα-negative breast cancer. However, further studies with large number of tumours and breast cancer cell lines are required to validate the precise function of *TRPS1* gene in breast cancer.

## Abbreviations

TRPS1: Tricho-rhino-phalangeal syndrome I; EMT: Epithelial to mesenchymal transition; ER: Estrogen receptor; IMP3: Insulin-like growth factor II (IGF-II) mRNA-binding protein 3; TNBC: Triple negative breast cancer; SMA: Smooth muscle actin.

## Competing interests

The authors declare that they have no competing interests.

## Authors’ contributions

PS and GZ conceived and designed the overall study. PS performed IHC and wrote the manuscript. HZ and XJ performed histological analysis and participated in the collection of data. JH and WL performed IHC manual assessment and analysis. MJ analysed the data and did the statistical data analyses. All authors read and approved the manuscript for publication.

## References

[B1] Cancer Genome Atlas N Comprehensive molecular portraits of human breast tumours Nature 2012 490 61 70 23000897 10.1038/nature11412 PMC3465532

[B2] Preat F Simon P Noel JC Differences in breast carcinoma immunohistochemical subtypes between immigrant Arab and European women Diagn Pathol 2014 9 26 24495621 10.1186/1746-1596-9-26 PMC3915228

[B3] Wang S Li H Wang J Wang D Expression of microRNA-497 and its prognostic significance in human breast cancer Diagn Pathol 2013 8 172 24143964 10.1186/1746-1596-8-172 PMC4015750

[B4] Zhang Q Zhang Q Cong H Zhang X The ectopic expression of BRCA1 is associated with genesis, progression, and prognosis of breast cancer in young patients Diagn Pathol 2012 7 181 23276146 10.1186/1746-1596-7-181 PMC3541118

[B5] Radvanyi L Singh-Sandhu D Gallichan S Lovitt C Pedyczak A Mallo G Gish K Kwok K Hanna W Zubovits J Armes J Venter D Hakimi J Shortreed J Donovan M Parrington M Dunn P Oomen R Tartaglia J Berinstein NL The gene associated with trichorhinophalangeal syndrome in humans is overexpressed in breast cancer Proc Natl Acad Sci U S A 2005 102 11005 11010 16043716 10.1073/pnas.0500904102 PMC1182410

[B6] Chen JQ Litton J Xiao L Zhang HZ Warneke CL Wu Y Shen X Wu S Sahin A Katz R Bondy M Hortobagyi G Berinstein NL Murray JL Radvanyi L Quantitative immunohistochemical analysis and prognostic significance of TRPS-1, a new GATA transcription factor family member, in breast cancer Horm Cancer 2010 1 21 33 21761348 10.1007/s12672-010-0008-8 PMC10358063

[B7] Momeni P Glockner G Schmidt O Von Holtum D Albrecht B Gillessen-Kaesbach G Hennekam R Meinecke P Zabel B Rosenthal A Horsthemke B Ludecke HJ Mutations in a new gene, encoding a zinc-finger protein, cause tricho-rhino-phalangeal syndrome type I Nat Genet 2000 24 71 74 10615131 10.1038/71717

[B8] Malik TH Shoichet SA Latham P Kroll TG Peters LL Shivdasani RA Transcriptional repression and developmental functions of the atypical vertebrate GATA protein TRPS1 EMBO J 2001 20 1715 1725 11285235 10.1093/emboj/20.7.1715 PMC145487

[B9] Napierala D Sam K Morello R Zheng Q Munivez E Shivdasani RA Lee B Uncoupling of chondrocyte differentiation and perichondrial mineralization underlies the skeletal dysplasia in tricho-rhino-phalangeal syndrome Hum Mol Genet 2008 17 2244 2254 18424451 10.1093/hmg/ddn125 PMC2710999

[B10] Piscopo DM Johansen EB Derynck R Identification of the GATA factor TRPS1 as a repressor of the osteocalcin promoter J Biol Chem 2009 284 31690 31703 19759027 10.1074/jbc.M109.052316 PMC2797240

[B11] Chang GT Steenbeek M Schippers E Blok LJ Van Weerden WM Van Alewijk DC Eussen BH Van Steenbrugge GJ Brinkmann AO Characterization of a zinc-finger protein and its association with apoptosis in prostate cancer cells J Natl Cancer Inst 2000 92 1414 1421 10974077 10.1093/jnci/92.17.1414

[B12] Stinson S Lackner MR Adai AT Yu N Kim HJ O’Brien C Spoerke J Jhunjhunwala S Boyd Z Januario T Newman RJ Yue P Bourgon R Modrusan Z Stern HM Warming S de Sauvage FJ Amler L Yeh RF Dornan D TRPS1 targeting by miR-221/222 promotes the epithelial-to-mesenchymal transition in breast cancer Sci Signal 2011 4 ra41 21673316 10.1126/scisignal.2001538

[B13] Chang GT Jhamai M Van Weerden WM Jenster G Brinkmann AO The TRPS1 transcription factor: androgenic regulation in prostate cancer and high expression in breast cancer Endocr Relat Cancer 2004 11 815 822 15613454 10.1677/erc.1.00853

[B14] Hong J Sun J Huang T Increased expression of TRPS1 affects tumor progression and correlates with patients’ prognosis of colon cancer BioMed Res Int 2013 2013 454085 23762846 10.1155/2013/454085 PMC3677607

[B15] Hu J Su P Jia M Wu X Zhang H Li W Zhou G TRPS1 expression promotes angiogenesis and affects VEGFA expression in breast cancer Exp Biol Med (Maywood) 2014 239 423 429 24595984 10.1177/1535370214523904

[B16] Gai Z Zhou G Itoh S Morimoto Y Tanishima H Hatamura I Uetani K Ito M Muragaki Y Trps1 functions downstream of Bmp7 in kidney development J Am Soc Nephrol 2009 20 2403 2411 19820125 10.1681/ASN.2008091020 PMC2799168

[B17] Itoh S Kanno S Gai Z Suemoto H Kawakatsu M Tanishima H Morimoto Y Nishioka K Hatamura I Yoshida M Muragaki Y Trps1 plays a pivotal role downstream of Gdf5 signaling in promoting chondrogenesis and apoptosis of ATDC5 cells Genes Cells 2008 13 355 363 18363966 10.1111/j.1365-2443.2008.01170.x

[B18] Thiery JP Epithelial-mesenchymal transitions in development and pathologies Curr Opin Cell Biol 2003 15 740 746 14644200 10.1016/j.ceb.2003.10.006

[B19] Thiery JP Epithelial-mesenchymal transitions in tumour progression Nat Rev Cancer 2002 2 442 454 12189386 10.1038/nrc822

[B20] Kalluri R Weinberg RA The basics of epithelial-mesenchymal transition J Clin Invest 2009 119 1420 1428 19487818 10.1172/JCI39104 PMC2689101

[B21] Thiery JP Acloque H Huang RY Nieto MA Epithelial-mesenchymal transitions in development and disease Cell 2009 139 871 890 19945376 10.1016/j.cell.2009.11.007

[B22] Kang Y Massague J Epithelial-mesenchymal transitions: twist in development and metastasis Cell 2004 118 277 279 15294153 10.1016/j.cell.2004.07.011

[B23] Blanco MJ Moreno-Bueno G Sarrio D Locascio A Cano A Palacios J Nieto MA Correlation of Snail expression with histological grade and lymph node status in breast carcinomas Oncogene 2002 21 3241 3246 12082640 10.1038/sj.onc.1205416

[B24] Prasad CP Rath G Mathur S Bhatnagar D Parshad R Ralhan R Expression analysis of E-cadherin, Slug and GSK3beta in invasive ductal carcinoma of breast BMC Cancer 2009 9 325 19751508 10.1186/1471-2407-9-325 PMC2753637

[B25] Hsu HP Shan YS Jin YT Lai MD Lin PW Loss of E-cadherin and beta-catenin is correlated with poor prognosis of ampullary neoplasms J Surg Oncol 2010 101 356 362 20119975 10.1002/jso.21493

[B26] Kim K Lu Z Hay ED Direct evidence for a role of beta-catenin/LEF-1 signaling pathway in induction of EMT Cell Biol Int 2002 26 463 476 12095232 10.1006/cbir.2002.0901

[B27] Xiang L Su P Xia S Liu Z Wang Y Gao P Zhou G ABCG2 is associated with HER-2 expression, lymph node metastasis and clinical stage in breast invasive ductal carcinoma Diagn Pathol 2011 6 90 21943250 10.1186/1746-1596-6-90 PMC3191358

[B28] Su P Zhang Q Yang Q Immunohistochemical analysis of Metadherin in proliferative and cancerous breast tissue Diagn Pathol 2010 5 38 20565850 10.1186/1746-1596-5-38 PMC2906416

[B29] Sung R Kang L Han JH Choi JW Lee SH Lee TH Park SH Kim HJ Lee ES Kim YS Choi YW Park SM Differential Expression of E-Cadherin, beta-Catenin, and S100A4 in Intestinal Type and Nonintestinal Type Ampulla of Vater Cancers Gut and liver 2014 8 94 101 24516707 10.5009/gnl.2014.8.1.94 PMC3916694

[B30] Hammond ME Hayes DF Dowsett M Allred DC Hagerty KL Badve S Fitzgibbons PL Francis G Goldstein NS Hayes M Hicks DG Lester S Love R Mangu PB McShane L Miller K Osborne CK Paik S Perlmutter J Rhodes A Sasano H Schwartz JN Sweep FC Taube S Torlakovic EE Valenstein P Viale G Visscher D Wheeler T Williams RB American Society of Clinical Oncology/College Of American Pathologists guideline recommendations for immunohistochemical testing of estrogen and progesterone receptors in breast cancer J Clin Oncol 2010 28 2784 2795 20404251 10.1200/JCO.2009.25.6529 PMC2881855

[B31] Sethi S Sarkar FH Ahmed Q Bandyopadhyay S Nahleh ZA Semaan A Sakr W Munkarah A Ali-Fehmi R Molecular markers of epithelial-to-mesenchymal transition are associated with tumor aggressiveness in breast carcinoma Transl Oncol 2011 4 222 226 21804917 10.1593/tlo.10244 PMC3140009

[B32] Chen JQ Bao Y Lee J Murray JL Litton JK Xiao L Zhou R Wu Y Shen XY Zhang H Sahin AA Katz RL Bondy ML Berinstein NL Hortobagyi GN Radvanyi LG Prognostic value of the trichorhinophalangeal syndrome-1 (TRPS-1), a GATA family transcription factor, in early-stage breast cancer Ann Oncol 2013 24 2534 2542 23729783 10.1093/annonc/mdt190 PMC3784330

[B33] Ye Y Xiao Y Wang W Yearsley K Gao JX Barsky SH ERalpha suppresses slug expression directly by transcriptional repression Biochem J 2008 416 179 187 18588516 10.1042/BJ20080328 PMC2584332

[B34] Paruthiyil S Parmar H Kerekatte V Cunha GR Firestone GL Leitman DC Estrogen receptor beta inhibits human breast cancer cell proliferation and tumor formation by causing a G2 cell cycle arrest Cancer Res 2004 64 423 428 14729654 10.1158/0008-5472.can-03-2446

[B35] Lazennec G Estrogen receptor beta, a possible tumor suppressor involved in ovarian carcinogenesis Cancer Lett 2006 231 151 157 16399219 10.1016/j.canlet.2005.01.021 PMC1942069

[B36] Skliris GP Leygue E Curtis-Snell L Watson PH Murphy LC Expression of oestrogen receptor-beta in oestrogen receptor-alpha negative human breast tumours Br J Cancer 2006 95 616 626 16880783 10.1038/sj.bjc.6603295 PMC2360679

[B37] Jensen EV Cheng G Palmieri C Saji S Makela S Van Noorden S Wahlstrom T Warner M Coombes RC Gustafsson JA Estrogen receptors and proliferation markers in primary and recurrent breast cancer Proc Natl Acad Sci U S A 2001 98 15197 15202 11734621 10.1073/pnas.211556298 PMC65006

[B38] Fantauzzo KA Tadin-Strapps M You Y Mentzer SE Baumeister FA Cianfarani S Van Maldergem L Warburton D Sundberg JP Christiano AM A position effect on TRPS1 is associated with Ambras syndrome in humans and the Koala phenotype in mice Hum Mol Genet 2008 17 3539 3551 18713754 10.1093/hmg/ddn247 PMC2572698

[B39] Wuelling M Kaiser FJ Buelens LA Braunholz D Shivdasani RA Depping R Vortkamp A Trps1, a regulator of chondrocyte proliferation and differentiation, interacts with the activator form of Gli3 Dev Biol 2009 328 40 53 19389374 10.1016/j.ydbio.2009.01.012

